# Warfarin and atrial fibrillation: from ideal to real the warfarin *affaire*

**DOI:** 10.1186/1477-9560-12-5

**Published:** 2014-02-18

**Authors:** Mauro Molteni, Claudio Cimminiello

**Affiliations:** 1 Internal Medicine, Vimercate Hospital, Azienda Ospedaliera di Desio e Vimercate, Italy

**Keywords:** Atrial fibrillation, Warfarin, Real life, Anticoagulation therapy

## Abstract

Vitamin K Antagonists (VKAs) are widely used in clinical practice and nearly 1% of the entire population receives oral anticoagulation at least once in life. However, the rate of prescription of anticoagulation is low, compared to what it should be. No more than 50-60% of patients affected by atrial fibrillation (AF) receive anticoagulation. In the setting of AF, VKAs are safe and effective when properly managed, reducing stroke and systemic embolism by more than 60%. VKAs safety and effectiveness are closely related to the quality of anticoagulation (e.g. time in therapeutic range), and anticoagulation clinics offer the best management of anticoagulant therapy. However, a sizeable proportion of patients are managed elsewhere. In clinical practice, in the setting of AF, a low prescription rate of VKAs is frequently observed and this is due also to difficulties in managing laboratory monitoring and drug dose adjustment. The suboptimal management of therapy with VKAs leads to a lesser efficacy than that reported in clinical trials, and to an increase in adverse reactions. VKAs still remain the first and only available therapy for a number of diseases (e.g. valvular atrial fibrillation and mechanical prosthetic heart valves). Now, since approval of the new oral anticoagulants (NOAs), the choice of anticoagulant therapy in definite settings, such as stroke prevention in non-valvular atrial fibrillation (SPAF) or treatment of venous thromboembolism, has surely become more intriguing but also more problematic. In light of these new therapeutic options, we reviewed VKAs therapy, in the setting of atrial fibrillation, focusing on VKAs impact in real life. We analyzed the data about efficacy and safety of warfarin at three levels: clinical trial and real life, outside and inside anticoagulation clinics.

## Review

### Introduction

Since its unexpected discovery in 1933, warfarin and, more generally, vitamin K antagonists (VKAs) have been widely used in clinical practice. Their peculiar pharmacokinetics and their management induced the development of professionals and clinics dedicated to anticoagulation therapy, e.g. the anticoagulation clinics. A variable proportion of patients treated with VKAs are managed in anticoagulation clinics, and another sizeable proportion of patients are managed elsewhere, e.g. general practitioners, cardiologists, neurologists etc.

The choice of anticoagulant therapy, after approval of the new oral anticoagulants (NOAs), has surely become more intriguing but also more problematic. The efficacy and safety of warfarin in trials are hardly reproducible in clinical practice, because of its peculiar characteristics. When comparing warfarin to something else, this specific issue should be considered. Therefore, we reviewed warfarin therapy, focusing on stroke prevention in atrial fibrillation (SPAF), to describe the discrepancies existing between clinical trials and real world. We analyzed the data about efficacy and safety of warfarin at three levels: that of clinical trial and meta-analyses, to be defined as ideal, due to the optimal management and patient compliance at that level; that of real life, at the level of dedicated anticoagulation clinics, which involve a very high accuracy but cannot be extended to the entire population of eligible patients; and that of real life, where patients receiving warfarin are managed by their general practitioners or other specialists.

We performed our narrative review of literature by searching publications up to July 2013 in PubMed and review of relevant reference citations. The key words (observational study [Title]) OR (warfarin [Title]) OR (vitamin K antagonist [Title]) OR (anticoagulation clinic [Title]) OR (INR [Title]) OR (Time in Therapeutic Range [Title]) OR (atrial fibrillation [Title]) OR (real world [Title]) OR (current practice [Title]) OR (dabigatran [Title]) OR (rivaroxaban [Title]) OR (apixaban [Title]) were combined to search the database. The additional filters “controlled clinical trial” “meta-analysis” “review” “abstract available” “publication dates from 01-01-1988 to 30-06-2013”, “humans” and ”English language” were applied to the search results. We retrieved 3178 papers; 2878 were discarded as not relevant to the review. Of the 300 selected, 271 were not taken into consideration because they were review articles which were in any case used in searching reference citations relevant to the topic. Therefore 28 articles were selected and 20 additional papers were identified by screening the references of the 300 aforementioned articles. The present overview has been carried out by trying to take into account the quality level of mentioned studies a number of whom, however, have been conducted 15 or even 20 years ago and their methodology would be today hardly acceptable in the light of current quality standards.

### Efficacy and safety of warfarin in clinical trials and meta-analysis

Most of the knowledge on the efficacy and safety of warfarin, in the setting of atrial fibrillation, comes from clinical studies performed almost thirty years ago. Hart’s meta-analysis [1] outlined warfarin effectiveness, defining its ability to reduce the incidence of ischemic stroke and systemic embolism in patients affected by non-valvular atrial fibrillation. Hart and colleagues reviewed sixteen trials including nearly 10,000 patients, and adjusted-dose warfarin was compared both with placebo and aspirin. In six trials involving nearly 3,000 patients, oral vitamin K antagonists were compared with placebo in primary and secondary prevention. The median age of these patients was 69 and only 20% had experienced a previous stroke or TIA. The patients assigned to placebo had a net stroke incidence of 4.6% in primary prevention and 12.3% in secondary prevention. Warfarin reduced the incidence of stroke by 59% in the setting of primary prevention and by 68% in secondary prevention, and absolute risk reduction was respectively 2.7% and 8.4%. When only ischemic strokes were considered, warfarin prevented 65% of strokes. As far as bleeding complications were concerned, warfarin induced twice as many major bleedings than placebo. Warfarin also induced a statistically significant all cause mortality reduction: it was observed a 26% relative risk reduction in mortality corresponding to a 1.6% absolute reduction. This meta-analysis also compared VKAs with aspirin alone: five non-blinded randomized trials were considered, involving more than 2,800 patients. Adjusted-dose warfarin was associated with a 36% relative risk reduction of all strokes; when only ischemic strokes were considered the relative risk reduction was 46%. The discrepancy between all stroke and ischemic stroke risk reduction was largely attributable to the results of the SPAF II study [2] and especially to the excess of intracranial hemorrhages. In this study, the target therapeutic range of International Normalized Ratio (INR) was between 2 and 4.5, which is now considered as being supra-therapeutic. The incidence of intracranial hemorrhage was more than doubled in this study compared to other trials. When SPAF II trial was excluded from meta-analysis, the relative risk reduction for all strokes was 49%. Warfarin induced twice as many intracranial and major extracranial hemorrhages than aspirin and reduced mortality by a non-significant 8%.

Although methodological features and quality of the trials varied substantially and often were incompletely reported, the results of this meta-analysis were confirmed by a second meta-analysis, published in 2007 [3]. The same group of Hart and colleagues analyzed several studies involving about 28,000 patients and produced similar results to those obtained in the previous decade.

More recently, warfarin effectiveness and safety was demonstrated through direct comparison to anti-platelet therapy. The Active W Study [4] enrolled nearly 7,000 patients with AF who were randomized to adjusted-dose warfarin or to the combination of aspirin and clopidogrel. The study was prematurely stopped because of evidence of superiority of anticoagulation. Warfarin reduced relative risk of stroke by more than 30%, and was at least as safe as dual anti-platelet therapy. The BAFTA study [5] assessed the role of anticoagulation compared to aspirin alone in elderly patients with atrial fibrillation, and showed how warfarin halved the risk of stroke with a similar rate of major hemorrhages. Similar results were obtained by the previous SPAF trial [6], which found no difference in overall major bleeding between aspirin and warfarin. Furthermore, Hylek et al. [7] added information on warfarin efficacy. They studied incident ischemic stroke in a cohort of more than 13,500 patients with non-valvular atrial fibrillation and clearly demonstrated that good-quality anticoagulation (e.g. INR of 2 or greater) significantly reduced the risk of ischemic stroke, its severity and the risk of death. Aspirin efficacy was no different from that of poor-quality anticoagulation as far as the risk of death was concerned.

The influence of anticoagulation control over outcome was clearly evidenced by White and colleagues [8]. They reported a clear relationship between the time in therapeutic range (TTR) and the risk of death and vascular events: patients with a TTR less than 60% had higher rates of annual mortality and major bleeding compared to patients with good INR control [TTR >75%], respectively 4.2% vs 1.69% and 3.85% vs 1.58%; the difference remained statistically significant also when compared to patients in moderate control [TTR, 60-75%].

The direct comparison of warfarin to the new oral anticoagulants (NOAs), e.g. dabigatran [9], rivaroxaban [10] and apixaban [11] outlines the safety profile of these new drugs, whose main advantage is to reduce intracranial hemorrhage. The NOAs, compared to warfarin, are also able to significantly reduce by nearly 10% overall mortality, although such a difference was not statistically significant in all the studies. In these trials, except in ROCKET-AF [10] with rivaroxaban, the warfarin whom the NOAs are compared to is a good quality warfarin with a 64% TTR. Rivaroxaban and dabigatran 150 mg bid had the same safety profile of warfarin while apixaban and dabigatran 110 mg bid were safer than warfarin. Furthermore, apixaban and dabigatran 150 mg bid significantly reduced the incidence of stroke and systemic thromboembolism. Although the new oral anticoagulants homogeneously reduced the risk of intracranial hemorrhage, rivaroxaban and dabigatran increased the incidence of gastrointestinal bleeding.

### Warfarin in the real life of anticoagulation clinics

The evidence coming from the results of the trials and meta-analysis aforementioned offers a safe and effective drug. The variability of warfarin effect, and the wide spectrum of drug interactions [12], implies frequent INR monitoring and dose adjustment. The challenging management of warfarin induced the development of professionals and clinics, dedicated to anticoagulation therapy, e.g. the anticoagulation clinics. Furthermore, algorithms for warfarin dose adjustment, for thrombotic and hemorrhagic risk, were created in order to help physicians in the anticoagulation management.

Anticoagulation clinics are dedicated to the management of anticoagulation, and this enables achievement of higher TTR; the higher the TTR, the safer the anticoagulation therapy. In 1996, the Italian federation of anticoagulation clinics, FCSA (*Federazione Centri Sorveglianza terapia Anticoagulante*), published a prospective cohort study on 2,745 consecutive patients treated with VKAs, studied from the start of their oral anticoagulation [13]. They achieved a median TTR of 68%; patients with low intensity anticoagulation performed an even better TTR, compared to patients treated with a high-intensity regimen. The rate of fatal and major bleedings, among these patients, was extremely low: respectively 0.25 and 1.1 per 100-patient-years of follow up. The rate of bleeding was significantly higher in the first 90 days of treatment, and in elderly patients (≥ 70 years-old). Bleeding rate was lower in patients who received anticoagulants because of venous thromboembolism. A more recent report of Italian FCSA [14] confirmed the safety of warfarin, and the low rate of major bleedings among elderly patients managed by anticoagulation clinics: the bleeding rate was 1.87 per 100-patient-years of follow up, with 0.55 intracranial hemorrhages. Nearly one fifth of all bleedings occurred in the first three months of treatment.

There are few direct comparisons on the quality of TTR between countries. The existing data addressing this issue come from the ISAM Study [15]. This was a retrospective, multi-centre cohort study, enrolling about 1,511 patients, randomly recruited from representative practices (routine medical care in the US, Canada, and France; anticoagulation clinics in Italy and Spain). Italy and Spain achieved higher levels of TTR compared to US, Canada and France. However, most of the Italian and Spanish patients were managed in anticoagulation clinics, while patients in the other countries were mainly managed in routine medical care. Anticoagulation clinics displayed a higher quality anticoagulation compared to routine medical care [16].

Anticoagulation clinics employed algorithms to simplify warfarin dose management and computerized algorithms were also validated for clinical use. The group of the Hamilton General Hospital [17], based on the experience of Detroit Henry Ford Hospital, formulated a simple two-step dosing algorithm which allowed a significant improvement of TTR. The employment of computerized algorithms [18] guaranteed a faster achievement of the steady state in the induction phase and the achievement of a higher TTR in the maintenance phase (71.2% vs 68.2%). Algorithms including pharmacogenomic data, e.g. polymorphisms of VKOR1 and CYP2C9 genes, have proved more accurate in predicting warfarin dosage [19]. Despite the demonstration of a more accurate prediction of warfarin dose, its cost-effectiveness is still debated [20] and international guidelines still do not suggest routine use of pharmacogenomics in anticoagulant therapy [21].

In order to simplify the management of anticoagulant therapy, a number of clinical trials evaluated the use of patient self-testing (PST) and patient self-management (PSM). A recent meta-analysis by Bloomfield et al. [22] included twenty-two studies that enrolled more than 8,400 patients. Fewer than 50% of potentially eligible patients successfully completed the training and agreed to be randomly assigned. PST and PSM patients had lower total mortality (Odd ratio, OR 0.79), lower risk for major thromboembolism (OR 0.58) and no increased risk for a major bleeding event. However the greatest pitfall of these studies is that most of the patients were Caucasian and most of all had a high level of education, which is not commonly found in real practice.

### Warfarin in real life outside anticoagulation clinics

The majority of patients treated with VKAs are managed in routine medical care, outside anticoagulation clinics. A recent survey, conducted through interview of Italian general practitioners, reveals that no more than a third of all the VKA-treated patients are managed in anticoagulation clinics [23]. This is not a minor issue because the efficacy and safety of anticoagulation depend on the management setting. Answell et al. [15] in ISAM study, highlighted the different performance, in terms of TTR, between these two settings. Van Walraven and colleagues of the Ottawa Health Research Institute [24] reviewed all published randomized or cohort studies that measured international normalized ratios (INRs) serially in VKA treated-patients. This meta-analysis comprised more than 50,000 patients, most of whom had been treated in anticoagulation clinics; only 24.4% had been treated in community practices. The median TTR was 63,6%; the only factors related to TTR were study setting, self management and drug (acenocoumarol vs warfarin). TTR in clinical trials was 12.2% higher than that of community practice and the difference between community practice and anticoagulation clinics was statistically significant with a 8.3% difference in TTR. The patients treated with warfarin in community practice had a median TTR of only 50%.

TTR is directly related to outcome [7]. In the ACTIVE W study [25], clinical benefit was strictly related to time in therapeutic range. A TTR less than 58% did not allow to detect any advantage of warfarin over dual anti-platelet therapy, in terms of clinical efficacy. RE-LY trial, comparing dabigatran and warfarin in SPAF, clearly showed how the advantage of dabigatran over warfarin was directly related to TTR performance [26]. When TTR was over 72%, dabigatran did not perform any better than warfarin.

Clinical trials and anticoagulation clinics performances, in terms of TTR, are hardly reproducible in community settings. A recently published survey provided us with an analysis of the Italian landscape [27]. Degli Esposti et al. analyzed the database of three big local health agencies, encompassing more than 770,000 persons. They identified 10,833 patients affected by atrial fibrillation and treated with VKAs, and measured their adherence to treatment, and the quality of anticoagulation. Median TTR of naïve patients was 47.9%, while patients in long-term anticoagulation had a median TTR of 56%. Even the patients in long-term stable therapy, who displayed a good adherence to warfarin, had a TTR no higher than 60%.

This poor quality anticoagulation translates into a reduced effectiveness. Indeed real practice analysis does not reproduce the 62% reduction of stroke incidence observed in Hart’s meta-analysis [1]. Caro et al. [28], compared Canadian VKA-treated outpatients for SPAF with patients without any antithrombotic treatment and observed a dismal 30% reduction of stroke. Darkow and colleagues [29] selected 12,359 patients with atrial fibrillation from a large healthcare system. More than 61% of these patients did not receive warfarin or VKAs. The Authors found that the relative risk reduction of vascular events in warfarin-treated patients was 22% as compared to those not treated with warfarin. In a wide group of Medicare patients, Birman-Deynch et al. [30], observed a 35% relative risk reduction in VKA-treated patients as compared to those receiving no anti-thrombotic therapy. These studies should be compared with a similar real life survey, the ATRIA Study [31], conducted on 13,428 patients, most of whom were treated in anticoagulation clinics. Relative risk reduction was found to be nearly 50% with a net benefit in overall survival. Although the results of ATRIA Study [31] are not directly comparable to those of Darkow [29] and of Birman-Deynch [30], the importance of different setting where the anticoagulation is managed seems to be evident in terms of effectiveness.

Real practice differs from clinical trials and from anticoagulation clinics also from the safety point of view. Gomes et al. [32] recently published data regarding the safety of oral anticoagulant therapy in more than 125,000 patients. The rate of major bleeding in real life was more than double than that reported in anticoagulation clinics [13,14]. Bleeding risk was directly proportional to age and CHADS_2_ score. Two thirds of bleedings were gastrointestinal, and the fatality rate was 14%, while intracranial hemorrhages had a much higher mortality (e.g. 42%). No difference in TTR was observed between patients with bleeding and those without.

All these dilemmas and troubles translate into a very low rate of prescription of VKAs, thus reducing the benefit of a very powerful therapy. Real life data surveys show that the rate of warfarin prescription is low. The Euro Heart Survey on Atrial Fibrillation [33], conducted by the European Society of Cardiology, observed a prescription of anticoagulation that was no higher than 60% and paroxysmal atrial fibrillation was negatively related to prescription. A recently survey of Italian Cardiology and Internal Medicines [34], showed a similar rate of prescription of anticoagulation: advanced age and paroxysmal atrial fibrillation were negatively associated to prescription. Waldo et al. [35], reported a rate of prescription of 55% and found that advanced age and paroxysmal atrial fibrillation negatively influenced warfarin prescription. Surveys among general practitioners found an even lower prescription [36]. Although more than 90% of patients had a moderate or high risk of stroke, less than one third of these patients were treated with oral anticoagulants. Furthermore, oral anticoagulation therapy was “lost in translation” [37]: after 12 months, only 42% of the patients were still receiving warfarin and after two years the percentage of patients still taking anticoagulants dramatically reduced to 28%.

The two most frequently given reasons against prescription, were the risk of falls and a previous hemorrhage. These elements and the perceived risk of bleeding are two of the main reasons which explain the discrepancy between warfarin indication and prescription [38]. As far as the risk of falls is concerned, more than 20 years ago Man Son Hing et al. [39] outlined how ephemeral the risk of falls is and that it should not even be considered during anticoagulation prescription. During these last years a number of scores have been produced in order to weigh the hemorrhagic risk. Despite their low sensitivity and specificity [40,41], the European Society of Cardiology advices to use these scores [42]. A high hemorrhagic risk should not prevent warfarin prescription but should focus medical attention on the patient.

An indirect proof of the discrepancy between warfarin performance in clinical trial and in common practice comes from the experience with the NOAs in real world. Both rivaroxaban and dabigatran in clinical trials were associated with a higher incidence of gastrointestinal hemorrhages as compared to warfarin [9,10]. The U.S. Food and Drug Administration (FDA) conducted a survey, called Mini-Sentinel [43], to monitor the bleeding complications of dabigatran. They recorded the bleeding complications of two matched cohorts of newly treated patients, by comparing warfarin-treated patients to dabigatran-treated ones. This survey showed how, in real life, warfarin caused more bleeding from the gastrointestinal tract compared to dabigatran. The rate of gastrointestinal bleeding in dabigatran arm was similar to that reported in RE-LY; bleeding rate in warfarin treated patients was higher than that observed in RE-LY. A Danish survey [44], in which dabigatran-treated subjects with AF were compared with a propensity-matched cohort of warfarin-treated patients, confirms the findings of FDA Mini-Sentinel. Dabigatran gave a 40% reduction in gastrointestinal bleeding, a 30 to 50% reduction in overall mortality and furthermore also myocardial infarction was significantly reduced with dabigatran, in contrast with the findings of the RE-LY study [9,45]. This effect, rather than being due to a better performance of dabigatran in real practice, seems to be related to a worse than expected effect of warfarin.

## Conclusions

### From ideal to real: what’s left?

Warfarin and the other VKAs are effective drugs. Although the NOAs are safe and effective, warfarin seems to ensure, in vitro, a greater suppression of normal haemostatic mechanisms because of a better inhibition of thrombin generation [46]. Indeed, such a marked activity on blood coagulation might be responsible for the higher incidence of intracranial bleeding and for the apparent better protection from myocardial infarction [47]. In addition, in the RE-LY study, in the quartile of AF patients who showed the highest levels of TTR, no advantage of dabigatran at a dose of 150 mg × 2 could be noticed as compared to warfarin [25].

Warfarin and VKAs have a unique pharmacokinetic profile: the variability of their effect requires a close monitoring and management which led to the development of anticoagulation clinics. The effect and the safety of warfarin is directly proportional to the time spent in therapeutic range [7] but the higher the TTR the higher the number of blood tests. The best results are related to the best quality of anticoagulation [8] but how many people can achieve the best control? How many people can be treated by anticoagulation clinics? There are not much data about this issue however, it is likely that - at least in Italy - no more the 30-40% of the VKA-treated patients are managed in anticoagulation clinics [23]. The quality of therapy in anticoagulation clinics is comparable to the one we met in clinical trials however, that is not the case for the rest of the patients. Median TTR of outpatients is far from being optimal and only in a few patient we can hardly get near to a 60% TTR [27].

The management of VKAs requires experience and dedication. In real life, warfarin has a safety profile which is very much different from that of the clinical trials [32]: many patients experience bleeding complications or a wide variability of INR values that often induce the patient to seek for medical attention. Budnitz et al. [48], indeed, reported an analysis of hospitalization and iatrogenic side effects underlining how warfarin use was associated to a high rate of hospital admittance. One third of all hospitalizations due to drug side effects are related to warfarin.

Warfarin and the other VKAs still remain the first and only available therapy for a sizeable proportion of diseases for which yet no real alternatives are available. VKAs management is challenging; anticoagulation clinics offer the safest and most effective management of these drugs. However, oral anticoagulation therapy is widely used and anticoagulation clinics cannot take care of all the patients treated with VKAs. Therefore most of the patients are managed elsewhere. This obviously translates into a worse TTR and to a loss of efficacy and safety in anticoagulant therapy, leading to complications, hospitalizations and increased mortality. All this turns into cost increase. The reproducibility of NOAs effect in real life seems to be promising. The easier management of NOAs could lead to an increase of use of anticoagulant therapy and thus to spread stroke prevention and to spare mortality and morbidity.

Warfarin and VKAs are effective drugs. Their peculiar characteristics make them hard to handle. Dedicated professionals should manage them but, also because of practical issue, where possible, they should be substituted by drugs whose management and pharmacokinetics are definitely more plain.

## Abbreviations

VKAs: Vitamin K Antagonists; AF: Atrial fibrillation; NOAs: New oral anticoagulants; SPAF: Stroke prevention in atrial fibrillation; INR: International normalized ratio; TTR: Time in therapeutic range; FCSA: Federazione centri sorveglianza terapia anticoagulante (Federation of Italian anticoagulation clinics); PST: Patient self-testing; PSM: Patient self-management; OR: Odd ratio; FDA: Food And Drug Administration.

## Competing interests

The Authors declare that they do not have any financial or non-financial competing interests.

## Authors’ contributions

The authors equally contributed in the conception and the design of the article. They have been equally involved in drafting the manuscript and revising it critically. CC has given final approval of the version to be published. Both authors read and approved the final manuscript.
